# Supplementary material for: ‘We do not rush to the hospital for ordinary wounds (suḷu tuvāla)’: A qualitative study on the early clinical manifestations of cutaneous leishmaniasis and associated health behaviours in rural Sri Lanka
Source: PLoS Negl Trop Dis. 2023 May 12;17(5):e0010939. doi: 10.1371/journal.pntd.0010939 (PMC10208456; doi:10.1371/journal.pntd.0010939)
Supplement: S1 Table — (PDF) [file pntd.0010939.s001.pdf]

S1 Table. Content of the participant experience reflection journal (PERJ)

Sub-Section	Questions
Basic demographic information	Name, Sex, Age, Village, Grama Niladhari Division, Medical Officer of Health area, Telephone number, the date PERJ received, the date PERJ returned, Researcher's contact details, Date/time period of first notice of symptoms, Date/time period of confirmation of CL by a medical officer, current status of CL (healed, currently receiving treatment, treatment has stopped midway)
Information for participants	Aim of conducting the study
	Instructions on completing the PERJ
Questions to elicit the patient journey of people with CL	Q1: Do you remember the day that you first noticed the lesion(s)? What happened on that day? Can you explain the things that you did first after noticing the lesion(s)?
	Q2: What did you feel on the day that you first noticed the lesion(s)? And what did you feel on the day when you were confirmed with the sandfly disease (Leishmaniasis)?
	Q3: Did you think that this condition should be medically treated? Was there someone who persuaded you to get treatments? Can you explain the things that you did, the places that you visited, the persons that you met, and the type of treatments that you took to treat this disease? (If you had any home remedies, it would be really important to mention that as well)
	Q4: What were the responses from society, from your family, from your relatives and friends, after you had been diagnosed with sandfly disease? Can you describe some of the positive and negative responses you received?
	Q5: What are the incidents, you faced (incidents where you felt relief or discomfort) when you visited different places and you met certain people during the journey of seeking treatments for this disease? (You can make your explanation about the following places that you may have visited)<br>I. Government hospital<br>II. Private medical center<br>III. Traditional or Ayurvedic treatment center<br>IV. Any other places you visited or any other person that you met
	Q6: What were the physical, psychological, and social changes that happened in your life after getting the disease (Both negative and positive)?

	Q7: Going to a clinic during day-to-day activities is not an easy task. So, we would like to know the difficulties that you faced there. Can you describe what you felt on the first day of your clinic? What did you think of the clinic? Were you happy to attend the clinic? What were the difficulties you encountered?
	Q8: You must have seen and talked to several patients with the disease when you went to the clinic. Are there any patients you can remember specifically? Can you tell us what you thought about those patients? What do they think about the disease? Have they mentioned anything about them being marginalized from society because of this illness?
	Q9: In general, we would like to know your thoughts on this sand fly disease. How is this disease usually known in rural areas? What are the causes of getting this disease? How severe is this disease? What is the opinion of the public about this disease? Likewise, anything can be written here. In short, we need to know what your thoughts regarding this disease are.
	Q10: Can you draw a flow chart or a diagram to depict your patient journey from noticing the symptoms for the first time until your current situation (healed, currently having treatment, or gave up the treatments midway)? We have given you an example. The flow chart or the diagram is not required to be exactly like the example. You can draw it in your way.
	Q11: There might have been many incidents and people which encouraged you, helped you and as well as discouraged you along the patient journey. We would like to know about them as well. So, can you please write about them here.
