# Supplementary material for: ‘We do not rush to the hospital for ordinary wounds (suḷu tuvāla)’: A qualitative study on the early clinical manifestations of cutaneous leishmaniasis and associated health behaviours in rural Sri Lanka
Source: PLoS Negl Trop Dis. 2023 May 12;17(5):e0010939. doi: 10.1371/journal.pntd.0010939 (PMC10208456; doi:10.1371/journal.pntd.0010939)
Supplement: Attachment — Submitted filename: Response to reviewers comments.docx [file pntd.0010939.s002.docx]

Thank you for reviewing our manuscript titled ‘We do not rush to the hospital for ordinary wounds *(<suḷu tuvāla>)*’: Healthcare-seeking in cutaneous leishmaniasis in rural Sri Lanka’.

We appreciate the time spent and all the detailed comments from reviewers. We have addressed all the comments in the revised manuscript and describe the changes in the tables below.

**General response for all three reviewers**

We made a few additional revisions as a general response in light of the reviewers’ comments. This manuscript is about the self-reported early clinical manifestations of cutaneous leishmaniasis (CL), how people with CL interpret these manifestations and behave in a certain way which can influence their healthcare-seeking process. Describing the entire healthcare-seeking and associated factors in CL is beyond the scope of this manuscript. Therefore, we have changed the second part of the manuscript's title to reflect the focus of this study better. We changed the name of the data collection tool, previously known as ‘CL patient journey booklet’, into ‘Participant experience reflection journal’ (PERJ) because it better captures the methodological approach we used. For reasons of clarity and privacy of the participants, we modified the participant ID into a uniform letter-number combination (i.e., J01, J02…). Kindly note that all these additional changes are related only to the manuscript's terminologies to ensure clarity and uniformity. We did not make any changes associated with the methodological and analytic approach used within the manuscript.

We hope you find these changes suitable.

Sonali Dinushika Gunasekara, on behalf of all co-authors.

**Table 1**

| **Comments: Reviewer 1** | **Response from authors** |
| --- | --- |
| Gunasekara and co-authors present a study of self-described health seeking behavior related to cutaneous leishmaniasis in Sri Lanka. The manuscript is very well written, clear and logically structured. The topic is relevant as understanding motivations for people to (not) seek health care is crucial for information and education efforts aimed at reducing delays in the process and thereby reduce morbidity and the risk of onward transmission. | Thank you. |
| As with many social science studies, it is unclear how generalisable the insights are beyond the study population and especially the Sinhala Sri Lankan population. It is to be hoped that the group will in the future provide comparative discussions of the results obtained in different countries and thereby be able to better determine which aspects are more universal and which ones rather local culture dependent. | Thank you for the comment.  Our study reveals the characteristics of self-reported clinical presentation and related behaviours of people with CL in a disease-endemic rural region in Sri Lanka, which is vital for early detection and appropriate management of the disease. Qualitative research, like the one underpinning this paper, aims not to generalise but to uncover key experiences and issues around a particular topic. But the themes and subthemes identified in our study will be relatable to many in other cultural contexts (see the Discussion section)  Rather than generalising the specific findings, health-related behavioural patterns of CL and other infectious diseases, more broadly neglected health issues could widely depend on clinical manifestations and underlying social context. The study findings will help to understand this fact in similar study populations. We have elaborated this in line numbers 763-767 on page 28. |
| Overall, the manuscript is rather lengthy, and the authors are invited to consider shortening it/tightening up to reduce the word count. | Thank you for the comment. We accept that the manuscript is lengthy, but we had to elaborate some sections when addressing the reviewers’ comments. However, we shortened certain sections like results . |
| Line 148: what is a “death benevolent society”? | The Death benevolent societies in Sri Lanka are one of the main community-based societies where most community members gather regularly. This society is established in most localities in Sri Lanka. The primary focus of this society is to improve the community's welfare, particularly during a funeral, and the deceased person's family is supported with financial aid, food, and funeral activities. As the journal does not permit adding footnotes, we feel defining the Death benevolent society in the place where it is mentioned within the manuscript would disrupt the flow of the text. Therefore, we included a reference to rural organisations, including Death benevolent society (line number 202 on page 8). |
| Line 209 etc.: it is mentioned that 30 participants completed the patient journey booklet. How many potential participants were approached initially? | Thank you for the comment. Based on the principles of data saturation and pragmatic considerations of the COVID-19 period, we decided to approach 30 eligible participants using the snowball sampling technique. All 30 eligible participants consented to participate in the study. We have updated the section on ‘participant recruitment’ to include these details (line numbers 192-205 on pages 7 and 8). |
| Line 253: “the upper area of the hands” – do the authors mean arms rather than hands? “Between the shoulder and elbow” seems to refer to a part of the arms | Thank you for pointing this out. We rephrased the term ‘The upper area of the hands’ as ‘lateral aspects of the arms’ (line number 368 on page 14).  Also, the term ‘hand’ is rephrased as ‘arm’ in Table 1 on page 12. |
| Line 417 etc.: On multiple occasions, the authors mention the relatively long delay between the moment the participants became aware of the symptoms and the time they sought formal health care. It is unclear whether “seeking care” refers to the first time they presented at a health facility or the moment they received the correct diagnosis? | Thank you for pointing this out. Within the manuscript, we considered ‘seeking healthcare’ as the first time people with CL presented at a modern biomedical healthcare facility, and this has been indicated throughout the manuscript. |
| Also, it would be relevant to understand whether there were wrong diagnoses provided first before the final diagnosis of leishmaniasis had been established? In other words: How long was the delay between seeking health care for the first time and receiving the correct diagnosis and how common are wrong initial diagnoses (some comments seem to imply they exist)? | Thank you for this comment. This is a significant finding based on our data. Even though patients had visited a biomedical healthcare facility, there were instances of general practitioners failing to diagnose early manifestations as CL at the first visit (about one-third of the participants). We have now briefly discussed this evidence (line numbers 652-656 on pages 24-25). |

**Table 2**

| **Comments: Reviewer 2** | **Response from authors** |
| --- | --- |
| This is a strong paper about an important topic. The research methodology is strong, although some more details would be helpful. The data itself is rich and well presented. | Thank you. We included more details in the methodology section (Study design - page 6, participant recruitment - pages 7-8, data collection - pages 8-9, data analysis - page 9-10, research rigour and quality control - page 10, ethical considerations - pages 10-11). |
| Further interpreting and organising the findings, whether in line with a theoretical framework about healthcare seeking behavior, or even using a barriers and facilitators to care approach would deepen the potential to draw conclusions. | Thank you for the comment. Our study aimed to provide new insights around patient-reported early clinical manifestations of CL, patients’ interpretations, associated behaviours and how they affect healthcare-seeking, which has not been revealed before. We believe that describing the entire healthcare-seeking behaviour in CL in a theoratical framework is beyond the scope of this manuscript. Instead, we have used data on healthcare-seeking to describe the importance of early clinical manifestations of CL in early detection and case management. We have now emphasised the focus of this study under the objectives of this paper (line numbers 160-163 on page 6). We use the revised figures to explain the associations between study findings. |
| Finally, if possible, including a socio-economic and equity analysis would round out the interpretation within the context, which currently addresses primarily culture but no other contextual influences. | Thank you for the comment. We aimed to explore how patient-reported early clinical manifestations of CL, their interpretations and associated behaviours affect healthcare-seeking in CL within the underlying rural context. As describing the entire healthcare-seeking and its associated factors is beyond the scope of this manuscript. Even though we have not conducted a complete socio-economic and equity analysis considering the word count of the manuscript, we have analysed and interpreted the data around the socio-economic context where possible (i.e., line numbers 719-722 on page 26, line numbers 742-747 on page 27) |
| Methods: The qualitative research design is strong – my overall recommendation is to start with one or two sentences summarising the approach, to let the reader know what they are going to read details about. | Thank you. We added more details to the study design section to summarise our approach (line numbers 171-179 on page 6). |
| The objectives of the research with a clear testable hypothesis are not stated within the methods section. The objective of exploring individual perceptions of early CL manifestations and health-related behaviors, within the rural Sri Lankan context, is at the end of the introduction. This could instead be expanded into two or three clear objectives within the methods section. | Thank you for this comment.  Qualitative research does not start with a ‘testable hypothesis’ as in other research designs. Hence, we have not specified a hypothesis within the manuscript. We split the objectives to make them more straightforward and placed them in the introduction section in line with journal guidelines (lines 160-163 on page 6). |
| Study design section could benefit from more detail. | We added more details to the study design section (see lines 171-179 on page 06). |
| Reorganising the participant recruitment section could be helpful – first describing what people were eligible, how they were identified (perhaps in more detail than is currently presented), and then the criteria used to select among them. Think about giving enough detail so someone replicate the process. | Thank you for the comment. We have reorganised the participant recruitment section (line numbers 192-205 on pages 7 and 8). |
| Similarly, describe all methods related to booklets and all methods related to interviews, or add subheadings to differentiate between the two data collection methodologies. | Thank you for the comment. We have now split the two data collection methods and described all the details relevant to each method under the subheadings of ‘participant experience reflection journal’ (PERJ), and ‘post-PERJ interviews’ (line numbers 206-256 on pages 8 and 9). |
| Sample size – appropriate for a qualitative study like this | Thank you. |
| Codebook development and application is appropriate according to the methodology described. Please clarify if both coders each coded every transcript. I imagine that’s what you mean, but from the text it isn’t clear. | Thank you for pointing out this. Initially, two ECLIPSE researchers independently coded a sub-set of transcripts (around 10% of the total transcripts) and compared the generated codes. Later, one researcher coded the remaining transcripts, adding new codes to the coding scheme. We have elaborated this section under ‘data analysis’ (line numbers 267-271 on page 10). |
| I would describe the participatory approach used to develop the research and data collection tools earlier in the manuscript. It’s important from an equity perspective and crucial to the credibility and reliability of the data collected. | We have described the participatory approach used in the development of data collection tools earlier in the manuscript (line numbers 217-241 on pages 8 and 9). |
| How were field notes used during analysis, if at all? Or were they solely developed to ensure reflexivity? | Thank you for the comment. We used the field notes relevant to interviews to ensure reflexivity and interpret our findings in a context-specific manner. We included this on ‘Research rigour and quality’ (line numbers 277-278 on page 10). |
| Were findings shared with members of the CL community to validate findings and ensure responses were interpreted correctly? This is often considered a crucial step in qualitative research and is worth describing if it was done. | Thank you for pointing this out. Respondent validation was done during the interviews, and the study findings were shared with the community members. We described this procedure under the section ‘Research rigor and control’ (line numbers 278-282 on page 10). |
| No concerns about ethical or regulatory requirements. Add a description of how patients with limited literacy were consented, given that the methods says they were included. | Thank you for the comment. We added a description of how participants with poor literacy were included in the study under the ‘Ethical Considerations’ (line numbers 297-299 on pages 11). |
| Results: It would be helpful for interpretation of quotations (which are great!) to have a sentence before to introduce them, then a sentence after each quote to guide the reader in interpreting and integrating the information. | We provided an opening sentence before and a guiding sentence for interpretation after certain quotes, considering the length of the manuscript as well (i.e., in line numbers 332,338-339, 404-405, 411-412). |
| Concerns about the length of time that has passed? Recall bias for events so far in the past can be significant, should be included in the limitations. | Thank you for pointing this out. We have now included ‘Recall bias’ as a limitation of this study under ‘Discussion’ section (line numbers 767-772 on page 28). |
| Figure 3 has important information but could throw off the reader because the manner of presenting similar data shifts. Consistency in data presentation, whether via text, tables, or figures, could be helpful. | Thank you for the comment. We removed the quotations from Figure 3 and revised it to improve clarity. |
| Subdividing the long paragraph on treatments attempted by patients would be helpful, perhaps dividing between Western medicine and traditional medicine? Also mentioning if any of these treatments can be effective would be helpful for people not very familiar with CL. | Thank you for the suggestion. We have now subdivided the paragraph on ‘managing wounds at home’ (line numbers 546-593 on pages 20-22). The effectiveness of these self-management practices has also been described there. |
| Table 3 might be visualised another way to connect the data – perhaps showing which types of progression led to which actions (a flow chart perhaps, or some way to show that the second column contains many repeating actions) | We have now visualised the information included in Table 3 in a new figure (Figure 4). |
| Figure 4 is great conceptually and definitely needed to bring findings together, but a little confusing to interpret. Adding some labels and explaining what the different arrows mean and why they point to different boxes would be helpful. | Thank you for the comment. We have modified the Figure 4 (now Figure 5) and added a summary paragraph below the Figure to facilitate easy interpretation (Page 24, line numbers 633-638) |
| There are also conceptual frameworks related to healthcare seeking behavior that could be useful in the presentation and interpretation of findings. | Thank you for this comment. Primary focus of this study is to provide new insights into patient-reported early clinical manifestations of CL, patients’ interpretations, associated behaviours and how they affect healthcare-seeking. Describing the entire healthcare-seeking behaviour in CL is beyond the scope of this manuscript. Instead, we have used data on healthcare-seeking to explain the importance of early clinical manifestations of CL in early detection and case management. |
| Conclusions are supported by the data presented, but further analysis and interpretation could be useful. | Thank you for the comment. We have conducted further analysis and interpretations throughout the ‘Results’ and ‘Discussion’ sections which can be found with track changes. |
| Reasons behind the delay in care seeking – did you explore socio-economic reasons for delay? Such as distance or cost to receive care? Distrust of healthcare facilities? A further analysis, even in the discussion, that includes an equity of healthcare access and quality of care received are important. For example, from the data you presented it seems like a priority was returning to work, did this need to keep working influence care access? Framing this around barriers to and facilitators of care access could be another framing approach. | Thank you for the comment. Our focus in this paper is on the self-reported early clinical manifestations of CL, how people interpret these manifestations and behave in a certain way which can influence their healthcare-seeking process. Describing the entire healthcare-seeking process in CL is beyond the scope of this paper. However, we have added more information regarding access to healthcare (line numbers 742-747, page 27). |
| Authors discussed how data can be used to inform practice and policy. | Thank you. |
| Limitations section was not present and would be valuable. | We have now included the limitations of the study under ‘Discussion’ (line numbers 763-772 on page 28). |
| Much of the manuscript might benefit from a bit of reorganisation, ensuring like information is with like. For example, in the Introduction, describing transmission, then manifestations, then consequences of infection, then reasons to prioritise early diagnosis. Outlining then grouping information together by theme could help reduce the minor confusion when currently reading it. | Thank you for the suggestion. We have now reorganised the introduction section by changing the place where information is located (line numbers 99-136 on pages 4-5). New information has been added to the introduction section to improve clarity and to preserve the flow of the text (line numbers 145-147 on page 5). The long paragraphs in results section have been divided into short paragraphs (i.e., ‘managing wounds at home’ (line numbers 546-593 on pages 20-22) and modifications in figures such as Figures 3,4 and 5 have been made to improve the clarity and comprehensiveness. |
| Adding details to the introduction about prevalence would be helpful, prevalence is described as “high” compared to other countries in the Asian subcontinent, numbers would be useful. | We have included the incidence of CL in a few other countries in the Asian subcontinent (line numbers 137-139 on page 5). |
| Tables and figures are needed and useful, but none add clarity to interpretation, which is often the hope with these tools, but instead require their own interpretation. Making them clearer would be a huge help to the reader. | We have now turned Table 2 into a Figure to preserve the pattern of similar data shifts. Figures 4 and 5 have been modified to improve clarity and comprehensiveness. |

**Table 3**

| **Comments: Reviewer 3** | **Response from authors** |
| --- | --- |
| This is very important research to better understand and develop site-specific interventions to improve early detection and treatment for CL. | Thank you. |
| Availability of data- clarify how one can access this data. | All the data underlying the described findings are included within the manuscript and supporting information files. A separate statement on data availability has been submitted to the online submission system of PLOS NTD and will be included in the manuscript when published. |
| Abstract: line 25 - "failure" to understand. Failure is a strong word and failure of who or what. Reconsider alternative wording. | Thank you for pointing out this. We have changed the sentence to emphasize the importance of knowledge on early manifestations, people’s perceptions and behaviours for preventing NTDs (line 25-26 on page 2). |
| Abstract: findings- include a sentence on the time gap | Thank you for the suggestion. We have now included the time gap between the notice of symptoms of CL and the first visit to a modern biomedical healthcare facility within the abstract (line numbers 55-57 on page 3). |
| The study design and methods are presented clearly and are well-suited to address the aim of the research. | Thank you. |
| It is not clear what pool of eligible pts was identified prior to purposively selecting 30 adults. How many CL past/current patients did the community help identify? | Thank you for the comment. Based on the principles of data saturation and pragmatic considerations of COVID-19 period we decided to approach 30 eligible participants (10 participants from each study site) using the snowball sampling technique and all the 30 eligible participants that we approached consented to participated in the study. During the initial fieldwork, community members helped us to identify at least 5-8 eligible participants from three study sites. And the rest of the eligible participants were identified via the CL patients who were introduced by the community members. The section on ‘participant recruitment’ has been modified including these details (line numbers 192-205 on pages 7 and 8). |
| Include how you accommodated illiterate pts under 'ethical considerations' | Thank you for pointing this out. We have now moved the section on how we accommodated less-literate participants into ‘Ethical considerations’ (line numbers 297-299 on pages 11). |
| The results are presented based on the 4 themes. Different methods of presenting the data are much appreciated - good choice of quotes, excerpts from booklets, and tables. | Thank you. |
| Line 224- "time" of onset of CL may not be the best term since no one can pinpoint a time of onset. The results presented are more about how and what they noticed. I would reconsider the use of 'time'. Possibly using 'awareness of onset of CL'? | Thank you for pointing this out and we agree with the comment. We have now modified the term ‘Time of onset’ into ‘The initial notice of the onset of CL’ (line number 326 on page 13). |
| "a symptom of worm infestation" (line 309) and "a change due to ageing" (line 321)- give some weight to how often these sub themes emerged. It appears to be the experience of only one pt for each. If they are only one, consider a sub theme that covers a few of these 'other' experiences. "A change due to ageing" sounds like it fits under "a normal skin change"- consider describing this case under that sub-theme. | Thank you for the comment. We have now included "A change due to ageing" under "a normal skin change" (line numbers 432-446, pages 17-18) and "a symptom of worm infestation" under ‘other interpretations’ (line numbers 501-510, page 19). |
| "Non-suspicion due to lack of awareness" (line 335)- This section has some information that could benefit being presented early in this section. Readers want to know earlier what the local term for CL is, its meaning and how is CL understood (or not) in this context. Consider moving before "a normal skin change" | We agree with this comment and moved some important information around local context of CL into line numbers 417-421 on page 17.The phrase ‘Non-suspicion due to lack of awareness’ has been modified as ‘Not suspecting the symptoms as CL’ (line number 511, page 19). |
| In line with the previous comment, you use the term 'sandfly disease' a few times and it’s not clear how much of this is part of the local understanding. Having this background gives some context to their health seeking choices. In line 327 it is said that "villagers knew about the disease"- how and what do they know? if that knowledge is there, the results do not discuss any pts that may have then suspected it was CL or sandfly disease. Line 337 says "most of the pts did not suspect their lesion to be CL ... " This implies that some/very few may have suspected it as CL/sandfly disease. Further at line 391, beliefs about eggs laid by the sandfly during the bite is part of the local understanding of CL/sandfly disease but again data is not presented on how, when, why any pts consider their symptoms as potentially being CL and if this prompts them seek formal health care. This needs clarity. | Thank you for the comment. Rather than assessing the community awareness of CL during our qualitative study, we focused on exploring local interpretations and behaviours of people with CL before they sought treatment. However, we realised that the study participants’ knowledge on CL was primarily limited to the mere awareness of the name of the disease, before they were diagnosed with CL. Even though they had heard the name of the disease, none of the study participants had suspected their early symptoms as CL due to several reasons. We have now described this aspect in line numbers 418-421 (page 17), 512-515 (page 19) and line numbers 534-537 (page 20). |
| Line 382- "used herbal mixtures prepared using medicinal plants"- if you have the data to briefly provide more context around who and/or the process of preparing herbal mixtures- is it done by pts themselves or do they go to an herbalist or shop? | Thank you for the comment. These herbal mixtures are prepared by the patients themselves using locally available medicinal plants, and we have now described this in line numbers 566, 568-569 on page 21. |
| Line 407- is an Ayurvedic hospital considered formal health care? If so, add at line 188 where you describe formal healthcare. But this definition at line 188 defines it as health facilities that have western medicine which I question if ayurvedic hospitals do. | Thank you for pointing this out. We understand the confusion that has been caused due to the definition of formal healthcare. As the only effective treatments for CL are available in modern biomedicine, now we have removed the term ‘formal healthcare’ from the manuscript, and it has been replaced with the term ‘modern biomedical healthcare’. This change has been made throughout the manuscript and can be found with track changes. |
| Line 401- correct "all most" to almost | We have corrected the term in line number 596 on page 22. |
| Include limitations to the study | We have now included limitations of the study under ‘Discussion’ in line numbers 766-775 on page 28. |
| Line 500- delete 'for' in affected for people's | We have now removed the term ‘for’ from the sentence. |
| Line 535- 'how local knowledge is produced'- As stated in results section, this did not come out clearly in the results. | We have now rephrased this section in ‘Discussion’ in line numbers 763-772 on page 28. |
| Include point 3 of recommendations in the author summary in the main paper recommendations. It is an important recommendation. | We have now included the relevant recommendation into ‘Discussion’ in line numbers 778-788 on pages 28-29. |
