# Supplementary material for: ‘We do not rush to the hospital for ordinary wounds (suḷu tuvāla)’: A qualitative study on the early clinical manifestations of cutaneous leishmaniasis and associated health behaviours in rural Sri Lanka
Source: PLoS Negl Trop Dis. 2023 May 12;17(5):e0010939. doi: 10.1371/journal.pntd.0010939 (PMC10208456; doi:10.1371/journal.pntd.0010939)
Supplement: Attachment — Submitted filename: Response to editorial comments.docx [file pntd.0010939.s003.docx]

**Response to editorial comments**

| **Editorial comments** | **Response from authors** |
| --- | --- |
| Figure Files:  While revising your submission, please upload your figure files to the Preflight Analysis and Conversion Engine (PACE) digital diagnostic tool, https://pacev2.apexcovantage.com. PACE helps ensure that figures meet PLOS requirements. To use PACE, you must first register as a user. Then, login and navigate to the UPLOAD tab, where you will find detailed instructions on how to use the tool. | We have uploaded all the figure files to PACE tool while revising the submission. Therefore, we ensure that all the figures meet the PLOS requirements. |
| Data Requirements:  Please note that, as a condition of publication, PLOS' data policy requires that you make available all data used to draw the conclusions outlined in your manuscript. Data must be deposited in an appropriate repository, included within the body of the manuscript, or uploaded as supporting information. This includes all numerical values that were used to generate graphs, histograms etc. | As we included all the data within the manuscript, we provided the following data availability statement during the submission process; ‘All the relevant data are within the manuscript and its supporting information files’ |
| Reproducibility:  To enhance the reproducibility of your results, we recommend that you deposit your laboratory protocols in protocols.io, where a protocol can be assigned its own identifier (DOI) such that it can be cited independently in the future. Additionally, PLOS ONE offers an option to publish peer-reviewed clinical study protocols. | We have not conducted a laboratory-based study. Therefore, the deposition of laboratory protocols is irrelevant to this manuscript. |
